# Performance of Online Somatic Cell Count Estimation in Automatic Milking Systems

**DOI:** 10.3389/fvets.2020.00221

**Published:** 2020-04-28

**Authors:** Zhaoju Deng, Henk Hogeveen, Theo J. G. M. Lam, Rik van der Tol, Gerrit Koop

**Affiliations:** ^1^Department of Farm Animal Health, Faculty of Veterinary Medicine, Utrecht University, Utrecht, Netherlands; ^2^Chair Group Business Economics, Wageningen University and Research, Wageningen, Netherlands; ^3^GD Animal Health, Deventer, Netherlands; ^4^Farm Technology Group, Wageningen University and Research, Wageningen, Netherlands

**Keywords:** somatic cell count, online-California mastitis test, udder health monitoring, on-farm screening tool, automatic milking machine, mastitis, dairy cow

## Abstract

Somatic cell count (SCC) is one of the most important and widely used mastitis diagnostics. For detecting (sub)clinical mastitis, online SCC related measurements are more and more used in automatic milking systems (AMS). Sensors such as an automated online California Mastitis Test (O-CMT) allow for high frequency screening of high SCC cows within a herd, which makes it potentially powerful to identify episodes of mastitis. However, the performance of O-CMT measurements, as compared to SCC determined in the laboratory (L-SCC), has only scarcely been described. The aims of this study were (1) to assess the agreement between the O-CMT measurement averaged over different time windows and the corresponding L-SCC measurements; (2) to determine the optimal time window for averaging O-CMT as compared to L-SCC; (3) to explore the added value of time-series of frequent O-CMT measurements in individual cow udder health monitoring compared to L-SCC measurements. Data were collected from 50 farms in 6 different countries that were equipped with AMS using O-CMT measurements and also performed regular L-SCC testing. We found that the overall concordance correlation coefficient (CCC) between O-CMT and L-SCC was 0.53 but differed substantially between farms. The CCC between O-CMT and L-SCC improved when averaging O-CMT over multiple milkings, with an optimal time-window of 24 h. Exploration of time series of daily O-CMT recordings show that this is an effective screening tool to find episodes of high SCC. Altogether, we conclude that although O-CMT agrees moderately with L-SCC, because of its high measurement frequency, it is a promising on-farm tool for udder health monitoring.

## Introduction

Mastitis is one of the main diseases in dairy cattle and leads to economic losses, usage of antimicrobials, and reduced animal welfare (1, 2). Udder health monitoring programs including regularly measured somatic cell count (SCC) have been widely used as a first step to improve udder health (3). These monitoring programs create awareness of udder health problems which, combined with mastitis prevention plans, motivate farmers to change on-farm udder health management to decrease the incidence of mastitis (4, 5).

The SCC of composite cow milk, as part of a dairy herd improvement (DHI) program, is a key indicator for udder health monitoring in current practice (6) and is generally measured using flow cytometry-based laboratory techniques (7). This routinely measured SCC in the laboratory (L-SCC) has long been the standard for udder health monitoring (8). The collection and shipping of samples for SCC measurement, however, is costly and time consuming and therefore generally DHI testing is done only every 3–6 wk. More frequent measurements would allow for earlier diagnosis, but requires an on farm test that can be performed at low per sample costs. The online California Mastitis Test (O-CMT) measurement is an automated sensor for mastitis monitoring in dairy farms with an automatic milking system (AMS).

The principle of the O-CMT sensor evaluated in our study is based on an automated CMT by taking a fixed volume of well-mixed composite milk from a cow milking. The milk is mixed with a fixed volume of reagent, after which the viscosity of the mixture is measured. The measured viscosity is transformed into a value, expressed in cells/mL, based on a calibration curve (9). The O-CMT is not comparable to L-SCC in terms of test characteristics, missing data, calibration and quality control, but due to frequent measurements, it may serve as a useful on farm screening tool. Although a single O-CMT measurement may not be precise, averaging multiple O-CMT recordings within different time windows may be helpful in gaining precision. Thus, we assume frequently measured O-CMT averaged over a certain time window may yield a better correlation to L-SCC.

Until now, a number of comparisons between SCC measured on farm and L-SCC have been published (10–15). Due to the characteristics of the gelling process of the mixture, the agreement between O-CMT and L-SCC was found to be poorer in low SCC ranges (<200,000 cells/mL; 9), while higher ranges of SCC (> 500,000 cells/mL) show a fair to good correlation (12). Hence, the performance of the O-CMT likely depends on the udder health situation of the farm. However, the performance of O-CMT in a large number of herds with varying udder health status is unknown and thus the practical value of this frequent O-CMT measurements in the field is unclear. Therefore, the aims of this study were (1) to assess the agreement between O-CMT measurements in different time windows and L-SCC measurements under field conditions; (2) to determine the optimal time window for averaging O-CMT as compared to L-SCC; (3) to explore the added value of time-series of frequent O-CMT measurements in individual cow udder health monitoring compared to L-SCC measurements.

## Materials and Methods

### Data Collection

Routinely collected O-CMT data from January 1st, 2015 to April 29th, 2016 from AMS farms having an O-CMT sensor system produced by Lely Industries N.V. (Maassluis, the Netherlands) and the DHI milk production recording data from the same farms over the same period were provided by Lely Industries N.V. Details of the data collection can be found in Jensen et al. (16). The data consisted of the rough, non-validated data that farmers also use. In all datasets, country and farm identifications were transformed to non-traceable identifications by Lely because of privacy concerns. The AMS data consisted of country and farm identification, cow identification, parity, calving date, date and time of milking and O-CMT measurement. The default measurement frequency of O-CMT was every third milking. When a cow had a high SCC (>200,000 cells/mL), the measurement frequency became higher. Farmers were advised to calibrate the sensor twice per year using standardized cow milk sample provided by Lely. The DHI data consisted of farm identification, cow identification, DHI test date and L-SCC. The L-SCC were tested in different laboratories, depending from which country the farm was. Because of the position of those laboratories in the milk payment scheme, the laboratories were certified (ISO 13366-1) to ensure the quality of measurements. This study was carried out in accordance with the commitments contained in the Basel Declaration and adhered to the General Data Protection regulations of the European Union. As no animal experiments were performed, no ethical approval was required for this study.

### Data Preparation

In the dataset we observed a small proportion (0.009%) of O-CMT being from milkings with an extremely low milk yield (<1 kg). Incomplete milkings with O-CMT might occasionally be present in our dataset. The raw dataset contains 7,427,010 records and was cleaned as follows:

1. records (*n* = 95,669) with composite milk yield per milking <1 kg were deleted;
2. records (*n* = 153,735) within 7 days after calving were deleted because of the confounding effect of early lactation on the SCC;
3. records with no O-CMT values (*n* = 4,527,244) or O-CMT = 1,000 cells/mL (*n* = 39,455) were deleted; The latter records were deleted, because the sensor automatically transforms all O-CMT ≤ 1,000 cells/mL to 1,000 cells/mL;
4. records from cows on L-SCC test dates when no L-SCC was available (*n* = 730) or with L-SCC ≤ 1,000 cells/mL (*n* = 377) were deleted;
5. records (*n* = 358,985) from cows with an L-SCC <2, 000 cells/mL were deleted;
6. records (*n* = 2,693) from farms with ≤ 100 L-SCC measurements were deleted;
7. records from 7 days before until 7 days after the L-SCC test dates were selected for each cow. Within these 15 days (7 days before and after DHI test date plus the DHI test day) period, only records with valid O-CMT value for every day were selected, which resulted in 1,816,144 deleted records.

The resulting cleaned dataset used for further analyses. After cleaning the dataset, all SCC values were log_10_-transformed for the purpose of obtaining approximate normal distribution.

### Assessment of Repeatability of O-CMT

Before the evaluation of agreement between the two tests, we assessed the repeatability of the O-CMT measurements. We defined an episode as being the period 48 h before and after an L-SCC test date. Consequently, the records within 48 h before and after the L-SCC test dates for each cow were selected from the cleaned dataset. Only episodes with ≥ 1 O-CMT measurements for every day were selected. A linear mixed regression model was constructed using the O-CMT measurements as dependent variable and episode, herd and cow as random effects. That way we were able to estimate the variance in O-CMT within each episode for each cow from every herd. Consequently, the intraclass correlation coefficient (ICC), calculated from the four variance components (episode, cow, herd, and the residual) extracted from this linear mixed model, represents the repeatability of O-CMT measurements (which equals to 1—the underlying “true” variation and the measurement error of O-CMT measurements within the episode).

### Agreement Between O-CMT and L-SCC

Concordance correlation coefficient (CCC) between two continuous measurements is one of the most commonly used methods to evaluate the agreement between two tests (17). In this study, we calculated the CCC between O-CMT and L-SCC to evaluate the measurement performance of single O-CMT and its averages calculated over multiple time windows.

#### Single Comparison

For the single comparison between O-CMT and L-SCC, all L-SCC and a randomly sampled O-CMT record per cow on the DHI test dates were selected. We first examined the CCC between the selected O-CMT records and the corresponding L-SCC records using the Bland-Altman plot (18) to display the relationship between O-CMT and L-SCC. Meanwhile, we calculated the CCC between these O-CMT and L-SCC records.

Because DHI test results only had a test date and no time stamp, for each DHI test date, there were possibly multiple O-CMT records. All of these O-CMT records were used in the CCC calculation with equal weight in determining the optimal time window that would result in the highest CCC between average of multiple O-CMT and L-SCC.

#### Time Window for Averaging Multiple O-CMT

To determine which time window, using the average of O-CMT measured within, resulted in the highest correlation between the O-CMT and the L-SCC, 7 time windows centered around the DHI test dates were created. Time windows were constructed as multiples of 24 h before and after the center of the DHI test date, leading to 7 time windows (spanning 24, 48, 72, 96, 120, 144, and 168 h). We first selected the records within the 168 h time window (168 h before and after the DHI test date) for each cow and each DHI test date from the dataset. The records within the 168 h time window for each L-SCC record of each cow were regarded as an episode. For each episode, the number of O-CMT measurements per day was counted. Episodes were included when they were from farms that had at least 100 episodes with ≥ 1 O-CMT measurement(s) on every day within the episode. For each episode, the average of O-CMT for each of the 7 time windows was calculated.

To calculate the CCC, a linear mixed model was applied using the lme function in the nlme package [version 3.1–142; (19)]. To calculate the overall CCC of all farms, the test method (binary variable: O-CMT or L-SCC) was included in the model as fixed effect; random herd and random cow effect were also included in the model. To calculate the individual farm level CCC, test method and individual cow were used as fixed effect and random effect, respectively, by using the epi.ccc function in epiR package [version 1.0–11; (20)]. The CCC between the average of multiple O-CMT within different time windows and L-SCC were calculated for 3 different ranges of L-SCC (L-SCC within 1,000–9,999,000 cells/mL, 100,000–1,500,000 cells/mL (the performance range of L-SCC), 200,000–9,999,000 cells/mL).

In addition to identify the optimal time window, we tried to find potential factors associated with the individual herd level CCC at the optimal time window using the available data. A linear regression model was built using the individual herd CCC as dependent variable and herd average parity, monthly herd geometric mean of L-SCC and monthly herd milk yield as independent variable. A full model, as well as a model using backward selection based on AIC, were fitted. All analyses were performed in R version 3.6.2 (21).

### Case-Wise Evaluation of O-CMT and L-SCC Measurements

The time window which resulted in the highest CCC in the previous analysis was used for calculating the moving averages for multiple O-CMT measurements over a longer time period. Four different O-CMT 24 h patterns were selected, which were representative of SCC patterns observed in field data. These selected O-CMT patterns were plotted along with the L-SCC measurements in the same time frame. In this way, the practical relevance of frequent O-CMT measurements in detecting high SCC episodes due to (sub)clinical manifestations of mastitis was illustrated.

## Results

### Descriptive Statistics

The descriptive statistics of the final dataset for the calculation of CCC between O-CMT and L-SCC are provided in Table 1. In total, 434,371 records from 4,829 cows at 50 farms in 6 countries were used in the analysis. Large differences in herd size were seen between farms and countries, with farms from country 2 and country 3 on average being larger than other farms. Overall, O-CMT values were higher than L-SCC values. All the herd average L-SCC values were below 200,000 cells/mL and only farms from country 2 had a herd average O-CMT higher than 200,000 cells/mL.

**Table 1:** General descriptive statistics of the farms in the cleaned dataset for SCC measured online (O-CMT) and SCC measured in the laboratory (L-SCC) and the data for calculating the correlation between both.

**Country**	**Total number** **of farms**	**Average number of cows per farm**	**Number of records**	**Farm geometric mean of L-SCC (×1,000 cells/mL)**	**Farm geometric mean of O-CMT on L-SCC test dates (×1,000 cells/mL)**	**Farm average CV^**a**^ of L-SCC**	**Farm average CV of O-CMT on L-SCC test dates per cow**	**Concordance correlation coefficient between L-SCC and single O-CMT one L-SCC test dates per farm**
1	1	36	1,787	75	195	0.38	0.32	0.518
2	19	114 (12–475)	9,644 (485–34,644)	165 (49–336)	213 (83–339)	0.45 (0.37–0.53)	0.53 (0.46–0.61)	0.658 (0.241–0.851)
3	9	150 (69–278)	11,231 (4,302–29,613)	141 (75–262)	198.2 (132–313)	0.44 (0.36–0.52)	0.52 (0.43–0.62)	0.592 (0.437–0.778)
4	1	59	12,170	74	102	0.7	0.58	0.479
5	13	68 (17–161)	8,800 (579–28,623)	155 (39–315)	165 (79–309)	0.49 (0.37–0.61)	0.62 (0.51–0.72)	0.578 (0.341–0.759)
6	7	46 (28–62)	3,142 (1,955–4,959)	92 (45–141)	146 (97–230)	0.41 (0.34–0.49)	0.40 (0.35–0.46)	0.421 (0.136–0.533)
Total	50	97 (13–282)	8,693 (480–32,723)	145 (38–317)	186 (78–326)	0.46 (0.19–0.79)	0.53 (0.29–0.86)	0.525 (0.142–0.787)

A total of 2,361,484 records from 7,788 cows in 52 farms in 6 countries were included in the cleaned dataset. The numbers with parenthesis are averages and parenthesis are 2.5–97.5% quantile.

a *Coefficient of variation*.

### Assessment of Repeatability of O-CMT

A total of 144,048 records from 14,504 episodes and 4,829 cows at 50 farms in 6 countries were used for the estimation of the repeatability of O-CMT measurements. The estimated ICC was 0.58, which suggests that 42% of the variance within the episode was due to the O-CMT measurement. However, it was not possible to distinguish the “true” variation between O-CMT measurements from measurement error of the O-CMT.

### Concordance Correlation Coefficient Between L-SCC and O-CMT

#### Single Comparison

In total, 29,008 O-CMT records of 4,829 cows in 50 farms from 6 countries could be linked to 29,008 valid L-SCC measurements on the same day.

**Figure 1:**
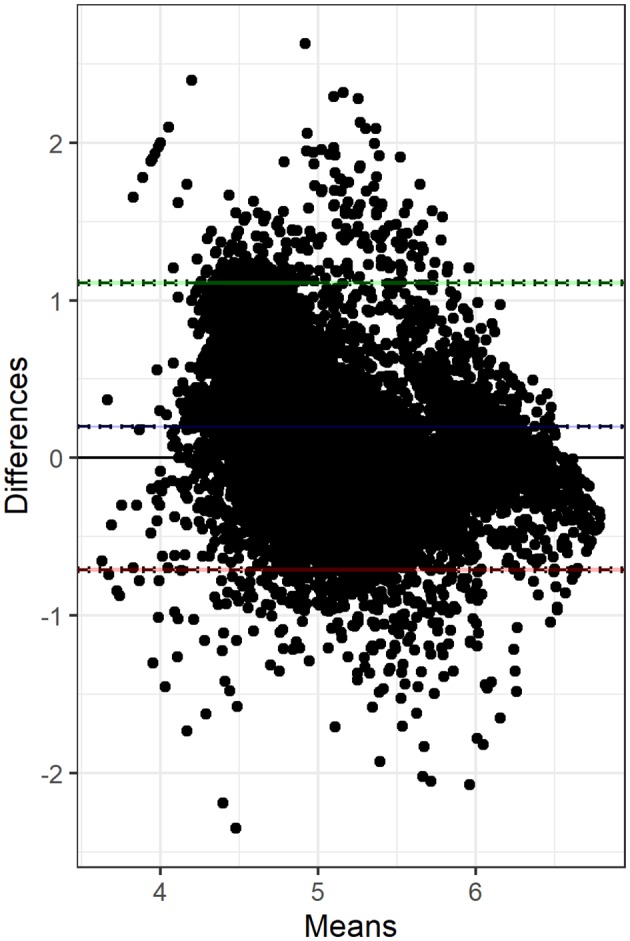
Bland-Altman plot displays the difference between single log_10_-transformed online CMT (O-CMT) values and log_10_-transformed laboratory measured SCC (L-SCC) against the average of both measurements on the DHI test days. Most of the records are within the limits of agreement. Overall, the differences between the two measurements are decreasing.

Figure 1 shows the Bland-Altman plot of the log_10_-transformed single O-CMT compared with the L-SCC measurement. The Bland-Altman plot suggests that the correlation between O-CMT and L-SCC is non-linear. The difference between these two measurements decreases in the high SCC area.

**Figure 2:**
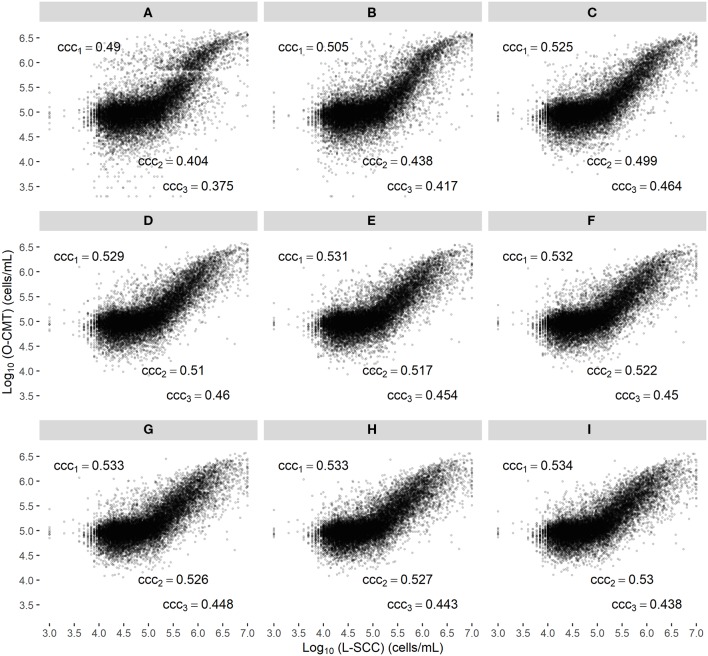
Scatter plot of the log_10_-transformed online CMT (O-CMT) values for randomly sampled one O-CMT records on DHI test dates against log_10_-transformed laboratory measured SCC (L-SCC) **(A)** and the average of multiple O-CMT within different time windows against L-SCC (**B–I**, corresponding to time windows from 0 to 168 h, increasing by steps of 24 h); ccc_1_ represents the overall concordance correlation coefficient between log_10_-transformed O-CMT and log_10_-transformed L-SCC, ccc_2_ is the concordance correlation coefficient with L-SCC within the range of 100,000–1,500,000 cells/mL and ccc_3_ is the concordance correlation coefficient with L-SCC in range of 200,000–9,999,000 cells/mL. Farms with ≥ 100 DHI tests with valid SCC results measured by O-CMT and L-SCC were included.

Figure 2A displays a scatter plot of the L-SCC and the randomly selected O-CMT measurement on each DHI test date per cow, and gives the CCC across several L-SCC ranges (1,000–9,999,000 cells/mL, 100,000–150,000 cells/mL, 200,000–9,999,000 cells/mL), showing that the agreement between L-SCC and O-CMT is better in the higher SCC regions but not necessarily with a higher CCC. The overall CCC between L-SCC and the average of O-CMT measurement within a 24 h time window was 0.53 (95% CI: 0.14–0.79).

#### Time Window for Averaging Multiple O-CMT

Figures 2B–I show that the CCC between averaged O-CMT within different time windows and L-SCC increased from Figures 2A–C (the 24 h time window) for all the 3 SCC ranges. The CCC in the 3 SCC ranges only increased marginally, when the time window was further expanded (Figures 2D–I). Therefore, we considered 24 h as the optimal time window to average the multiple O-CMT measurements in this study.

**Figure 3:**
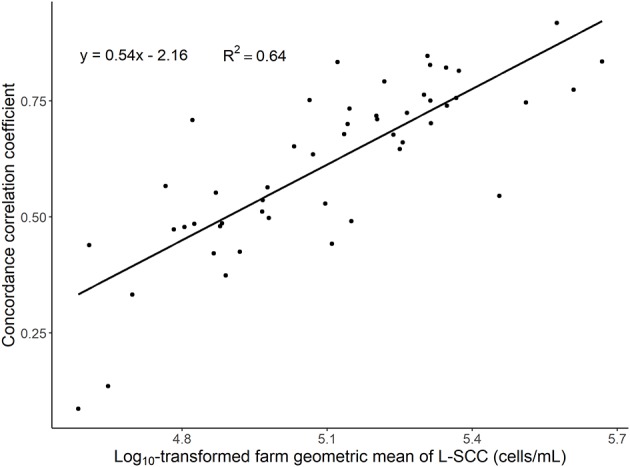
Scatter plot of concordance correlation coefficient between the average of multiple log_10_-transformed online measured CMT (O-CMT) values over a 24 h time window and log_10_-transformed laboratory measured SCC (L-SCC) against the log_10_-transformed geometric mean herd SCC per farm on 50 farms. The regression line has a beta estimate of 0.54 and the R-squared is 0.64.

We found substantial variation in CCC between O-CMT 24 h and L-SCC between farms. The farm-level CCC was positively related to the farm's geometric mean L-SCC (Table 2 and Figure 3).

**Table 2:** Correlation between online SCC estimation and the SCC measured in the laboratory in different studies.

**Study**	**SCC estimation method**	**Country**	**Number of** **farms**	**Number of AMS or SCC sensors**^**a**^	**Number of** **cows**	**Number of records**	**Correlation**
Casura et al. (10)	CMT^b^	NA^c^	1	NA	298	2,331	0.57
Leslie et al. (11)	CMT	Canada	1	2	140	1,000	0.71
Kamphuis et al. (12)	CMT	New Zealand	1	2	200	456	0.76
Mollenhorst et al. (13)	CMT	Netherlands	3	6	191	3,191	0.47
Neitzel et al. (14)	CMT	Germany	1	7	165	1,357	0.2–0.57
Sørensen et al. (15)	Flow cytometry	Denmark	7	>16	2,325	713,326	0.93^d^
Current study	CMT	6 countries	50	113	4,829	434,671	0.53^e^

a Automatic milking system or online somatic cell count sensor.

b SCC estimated based on the California mastitis test principle.

c Not found.

d The square root of R squared from regression using log-transformed L-SCC as dependent variable and log-transformed O-CMT as independent variable.

e *Concordance correlation coefficient between average of online-SCC within a 24 h time window and the SCC measured in laboratory*.

**Figure 4:**
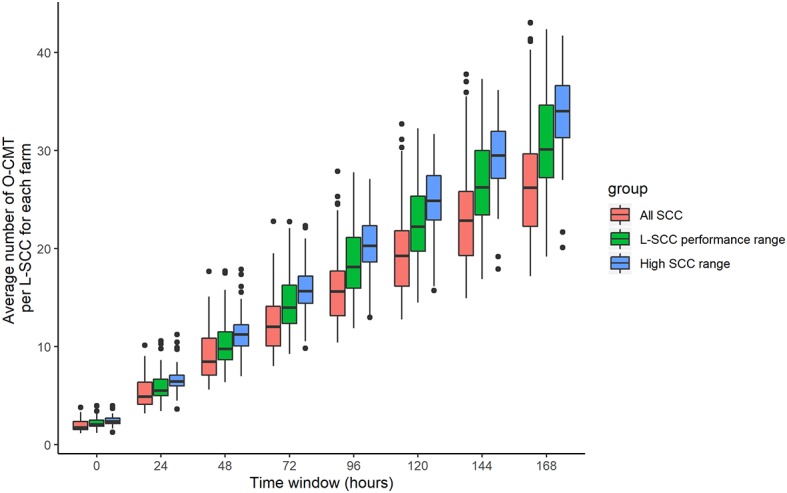
Farm average number of online CMT (O-CMT) values per SCC value measured in the laboratory (L-SCC) for all O-CMT and for L-SCC performance range (100,000–1,500,000 cells/mL) as well as high SCC range (> 200,000 cells/mL) separately for different time windows.

Figure 4 gives the number of O-CMT records per L-SCC record in different SCC ranges for the 7 time windows. It is obvious that the number of O-CMT measurements does increase with longer time windows. Moreover, it is also visible that more O-CMT measurements are made when O-CMT is higher (> 200,000 cells/mL). A 0 h time window averages about 2 O-CMT values, whereas a 24 h time window contains on average about 5 O-CMT records.

### Case-Wise Comparison of O-CMT With L-SCC Measurements

**Figure 5:**
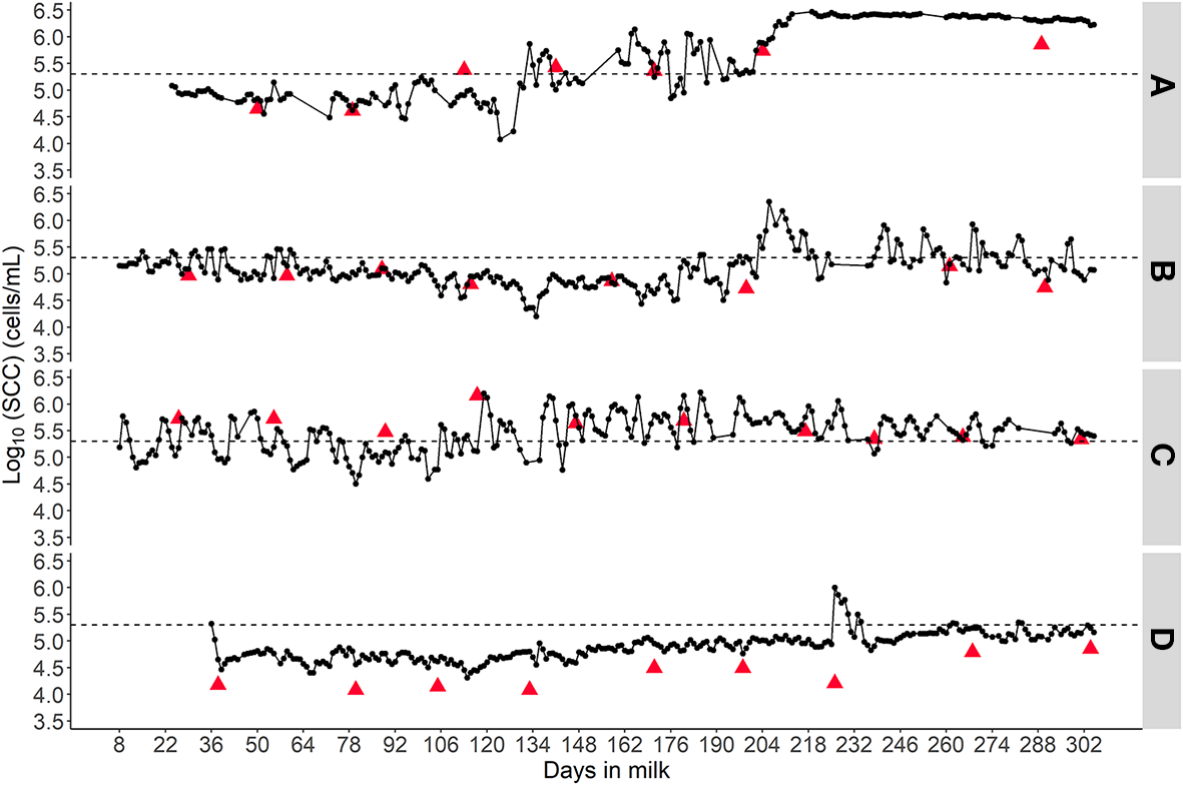
Four different SCC patterns to demonstrate the value of frequently measured online SCC in individual cow udder health monitoring. **(A)** Indicates a chronic intramammary infection; **(B)** suggests an infected udder that cured followed by a re-infection; **(C)** displays a cow likely with chronic IMI that shows a fluctuating SCC pattern and **(D)** probably is a healthy udder with one brief high SCC episode. The triangles represent laboratory measured SCC results and the dots connected by a line represent the online CMT measurements averaged over a 24 h time window. The dashed horizontal line represents 200,000 cells/mL.

Figure 5 displays 4 different SCC patterns from 4 different cows that were representative of our data. Overall, the O-CMT 48 h patterns were corresponding to the L-SCC patterns for each cow, Figure 5A shows a healthy udder before 130 DIM, with indication of two short (new) intramammary infection (IMI) occurring around 134 and 162 DIM, and of a chronic persistent IMI starting around 190 DIM; Figure 5B shows an IMI in early lactation that seemed to have cured between 64 and 180 DIM with indications of a new IMI in late lactation; Figure 5C presents an udder with a chronically persistent IMI with large variation in day-to-day O-CMT 48 h; Figure 5D indicates a healthy udder with a brief IMI in the late stage of lactation.

## Discussion

In this study, we aimed to evaluate the performance of O-CMT measurements in comparison to L-SCC. The value of O-CMT measurement is an estimation of SCC within ranges instead of an exact measurement of SCC (9). Hence the O-CMT values should be interpreted with caution. The overall CCC between O-CMT within a 24 h time window and L-SCC in 50 farms was 0.53 (95% CI: 0.14–0.79). The CCC increased most when averaging O-CMT over a 24 h time window. Our results suggest that frequent O-CMT measurement is a valuable on-farm tool for monitoring udder health of individual cows, despite the fact that a single O-CMT measurement may be less accurate than a single L-SCC measurement.

The data we used in this study consisted of rough, non-validated data, representative of how the data arises in practice. The samples from the O-CMT differed from the samples for the L-SCC. Besides that, it is clear that there is a lower level of quality control for the O-CMT measurements, for instance by non-optimal calibration procedures, in comparison to the L-SCC measurements. This may jeopardize the agreement between the two tests. Therefore, a direct comparison between the measurement systems in order to establish the preciseness of the O-CMT measurement is impossible with our data. However, by comparing the O-CMT measurements with the L-SCC measurements on milk from the same cow on the same day, we were able to provide insight in the practical usability of the O-CMT measurements.

Prior to the correlation analysis, we evaluated the repeatability of the O-CMT measurements within a 48 h time window assuming that the underlying SCC of a cow was stable within this 48 h time window (5 days). The repeatability, as represented by the ICC, was 0.58. Since natural daily variation in SCC exists, we consider the repeatability of O-CMT measurement to be acceptable within the period of 5 days.

We found an overall CCC between O-CMT and L-SCC of 0.53, which is in line with previous studies, that found values somewhat higher or lower than our estimate (Table 3). Previous studies, however, only used a small number of farms to assess these correlations. In our data, we found a large variation in CCC between farms. This between-farm variation was largely explained by the farm level L-SCC (Figure 3), likely due to the fact that the correlation is higher in the higher SCC ranges. In other words, the CCC might depend on the prevalence of high SCC cows on farms. As displayed in Figure 1, the difference between O-CMT and L-SCC was deceasing as the herd average L-SCC increases. There are several other reasons for the fact that the CCC between O-CMT and L-SCC differs between farms. First, although the sensor are “factory calibrated” and farmers are advised to perform the calibration twice per year, not all farmers may actually have done this. Neitzel et al. (14) reported a significant difference between sensor devices in measuring the O-CMT and showed that the Pearson's correlation coefficient between O-CMT and L-SCC was higher after calibration. These differences in calibration between farms or sensors will likely have led to an underestimation of the true overall correlation between both SCC measurement methods relative to using well-calibrated sensors.

**Table 3:** Estimates from linear regression model using the herd level concordance correlation coefficient between online CMT and SCC measured in laboratory as dependent variable and the herd average of monthly geometric somatic cell count (SCC_herd_), herd average parity (Parity_herd_), as well as herd average monthly milk yield (Milk yield_herd_) as independent variables.

**Variable**	**Estimate**	
	**Full model**	**Backward selection model**
Intercept	−2.33	−2.16
SCC_herd_	0.55	0.54
Parity_herd_	0.05	
Milk yield_herd_	0	

*Backward selection using AIC was applied for model selection. The full model included all the independent variables and the final model only with the variable remained in the model after model selection*.

Although CCC between O-CMT and L-SCC was rather not sufficient, we consider there are several reasons for this imperfect agreement between O-CMT and L-SCC. First, the O-CMT evaluated in our study uses a different technique, based on a CMT derived method to quantify the O-CMT whereas L-SCC actually counts the number of cells using flow cytometry. The online sensor has an algorithm that transforms the viscosity of the gel formed by DNA and test reagent, to an O-CMT value based on calibration against L-SCC. Thus, by definition, the indirectly measured single O-CMT is less accurate than a single L-SCC measurement. Second, the performance range of L-SCC (the range in which its accuracy is guaranteed) is 100,000–1,500,000 cells/mL (22) while we noticed that more than half of the L-SCC measurements in our dataset were <100,000 cells/mL. Measurements outside the range in which the two tests perform well-contributed substantially to the imperfect correlation between these two measurements (Figure 2A). The scatter plots in Figure 2 display weak S-shape, suggesting that the algorithm that transforms viscosity to an SCC value can be further optimized to better correlate to the L-SCC reference test. By adapting the transformation, the association between O-CMT and L-SCC can be made more linear, which should result in a higher (linear) correlation between the two. Lastly, although we did not evaluate that in this study, farmers may not re-fill the CMT reagent in time. Field experience learns this occurs and thus it may also affect the correlation between O-CMT and L-SCC.

With the availability of novel on-farm milk quality sensors, quality control of such measurements also has to be implemented on-farm. For decades, laboratories have calibrated their methods and compared their results, for instance by the use of ring trials. In contrast with these highly controlled laboratory systems, there is no systematic quality control system in place for automated on-line milk quality measurements. Since these on-farm milk quality systems become more and more important, it would be good if quality control programs for on-farm milk quality systems would be developed.

The L-SCC in our dataset were measured in different laboratories. Potentially there may be differences in L-SCC measurement between laboratories. However, data quality control in the laboratories for L-SCC measurements was assumed to be good because these laboratories are also involved in quality-based milk payment schemes and work under ISO certification (ISO13366-1). Meanwhile, by using a random herd effect in linear mixed models, potential laboratory effects were corrected for in the statistical modeling.

In Figure 2, we showed that the overall CCC between O-CMT and L-SCC in the range of 1,000–9,999,000 cells/mL, increased mostly at a 24 h time window. The overall CCC between O-CMT and L-SCC was increasing only slightly with longer time windows. There seems to be an optimum time window for averaging O-CMT, and we suggest 24 h as the optimal time window, in which the random error present in single measurements is strongly reduced, but the capacity to monitor infection dynamics over time is still acceptable.

The number of milkings with an O-CMT measurement per L-SCC measurement is substantially higher for high L-SCC (> 200,000 cells/mL) than for all SCC range, because of the algorithm that prescribes to measure O-CMT every milking after a high measurement is recorded, while the sensor only measures O-CMT every third milking in low SCC cows.

Figure 5 illustrates that the O-CMT measurements present the same trend as L-SCC, while giving more information on short high SCC episodes. This information is missed by L-SCC, given that DHI test is normally performed every 3–6 weeks, which limits the power of L-SCC in detecting high SCC episodes. Thus, O-CMT seems more valuable in individual cow udder health monitoring. In addition, there may be pathogen species that cause specific SCC patterns. De Haas et al. (23) found that clinical mastitis caused by *Escherichia coli* was significantly associated with a short peak in SCC while *Staphylococcus aureus* was significantly associated with longer increased SCC, whilst no clear patterns were found for *Streptococcus dysgalactiae* or *Streptococcus uberis*. Compared to traditional methods (e.g., bacteriological culturing), the use of frequent O-CMT measurements can serve as a cheap and fast on-farm screening method for mastitis. It is fully automated and can be executed for almost every milking. These characteristics make O-CMT and other on-line SCC measurement methods a suitable tool for on-farm individual udder health monitoring. The measurements may also be used to identify subclinical mastitis cases that warrant further diagnostics such as bacteriological culture to explicitly identify the mastitis-causing pathogens. Further research to link the O-CMT patterns to pathogen species would be useful and highly relevant to develop tailor-made treatment plans to further optimize treatment strategies and reduce antimicrobial usage. Our results show added value of O-CMT measurement, but to further quantify the added value of O-CMT in detecting high SCC episodes, more work is needed. Specifically, work should be carried out on algorithms to mine these intensively measured O-CMT for early detection of high SCC as well as to quantify long term udder health related effects (such as incidence rate of clinical mastitis, milk production, total antimicrobial usage) and the economic value of the use of O-CMT measurements.

## Conclusion

The overall concordance correlation coefficient between O-CMT and L-SCC of all farms was 0.66, and increases when the farm level SCC is higher. The average of multiple O-CMT measurements over a 24 h time window was found to provide an optimum between correlation between O-CMT and L-SCC and the capacity to capture udder health dynamics. The O-CMT measurement shows to be a promising on-farm tool for individual cow udder health monitoring, specifically because of its high measurement frequency.

## Data Availability Statement

The datasets generated for this study are available on request to the corresponding author.

## Ethics Statement

This study was carried out in accordance with the commitments contained in the Basel Declaration and adhered to the General Data Protection regulations of the European Union. As no animal experiments were performed, no ethical approval was required for this study.

## Author Contributions

ZD performed data analysis and wrote the manuscript. GK, TL, HH and RT designed the study. All authors interpreted the data, edited and approved the final manuscript.

## Conflict of Interest

TL was employed by the company GD Animal Health. The remaining authors declare that the research was conducted in the absence of any commercial or financial relationships that could be construed as a potential conflict of interest.

## Abbreviations

SCC: somatic cell count
O-CMT: online California mastitis test
L-SCC: somatic cell count determined in the laboratory
O-CMT 24 h: average of multiple online California Mastitis Test measurements within a 24 h time window
IMI: intramammary infection
DHI: dairy herd improvement.
